# Supplementary material for: Mortality Associated with Idiopathic Pulmonary Fibrosis in Northeastern Italy, 2008–2020: A Multiple Cause of Death Analysis
Source: Int J Environ Res Public Health. 2021 Jul 6;18(14):7249. doi: 10.3390/ijerph18147249 (PMC8305452; doi:10.3390/ijerph18147249)
Supplement: Supplementary file 1 [file ijerph-18-07249-s001.zip › ijerph-1238730-supplementary.pdf]

Marcon, A.; Schievano, E.; Fedeli, U. Mortality Associated with Idiopathic Pulmonary Fibrosis in Northeastern Italy, 2008–2020: A Multiple Cause of Death Analysis. *Int. J. Environ. Res. Public Health* **2021**

Supplementary Materials

Table S1. Annual percent change in mortality rates through the study period with 95% confidence intervals. Veneto region, 2008-2019.

	UCOD analysis<br>Annual percent change (95%CI)	MCOD analysis<br>Annual percent change (95%CI)
Males, age 40-74	-0.9 (-4.4, 2.7)	-1.5 (-4.3, 1.3)
Males, age 75-84	1.0 (-2.0, 4.2)	-0.8 (-3.0, 1.5)
Males, age ≥85	6.5 (2.0, 11.2)	3.5 (0.2, 6.9)
Females, age 40-74	-0.7 (-5.9, 4.8)	-1.7 (-5.7, 2.4)
Females, age 75-84	-0.7 (-4.4, 3.2)	-1.1 (-4.0, 1.9)
Females, age ≥85	-0.7 (-4.9, 3.7)	-1.8 (-4.9, 1.3)

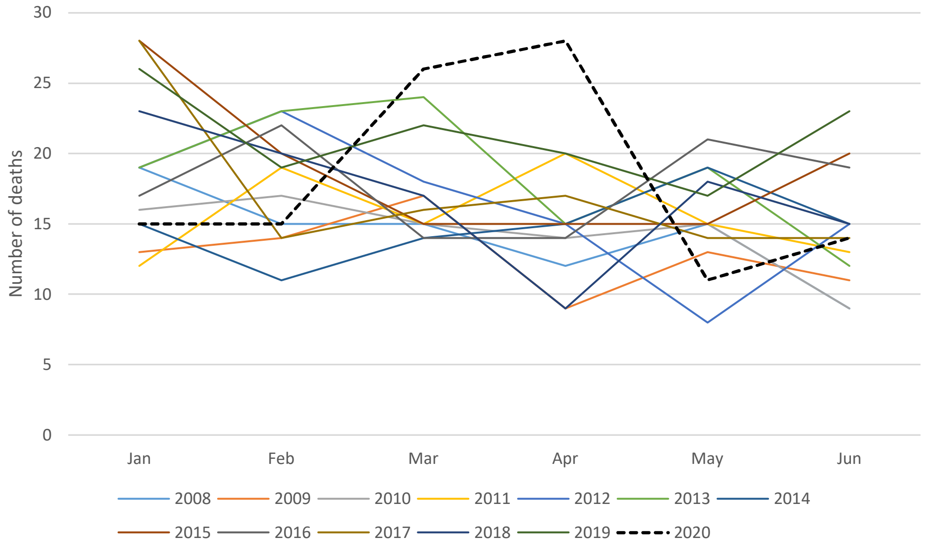

Figure S1. Number of death certificates with any mention of IPF by month in the first semesters of years 2008-2019 and provisional data from 2020.
